# Glycemic status, insulin resistance, and mortality from lung cancer among individuals with and without diabetes

**DOI:** 10.1186/s40170-024-00344-4

**Published:** 2024-06-20

**Authors:** In Young Cho, Yoosoo Chang, Eunju Sung, Boyoung Park, Jae-Heon Kang, Hocheol Shin, Sarah H. Wild, Christopher D. Byrne, Seungho Ryu

**Affiliations:** 1 Department of Family Medicine, Kangbuk Samsung Hospital, Sungkyunkwan University School of Medicine, 29 Saemunan-Ro, Jongno-Gu, Seoul, 03181 Republic of Korea; 2 Department of Family Medicine & Supportive Care Center, Samsung Medical Center, Sungkyunkwan University School of Medicine, Seoul, Republic of Korea; 3 Center for Cohort Studies, Total Healthcare Center, Kangbuk Samsung Hospital, Sungkyunkwan University School of Medicine, Seoul, 04514 Republic of Korea; 4 Department of Occupational and Environmental Medicine, Kangbuk Samsung Hospital, Sungkyunkwan University School of Medicine, Samsung Main Building B2, 250 Taepyung-Ro 2Ga, Jung-Gu, Seoul, 04514 Republic of Korea; 5 Department of Clinical Research Design & Evaluation, SAIHST, Sungkyunkwan University, Seoul, 06355 Republic of Korea; 6 Department of Preventive Medicine, Hanyang University College of Medicine, Seoul, Republic of Korea; 7 Usher Institute, University of Edinburgh, Edinburgh, UK; 8 Nutrition and Metabolism, Faculty of Medicine, University of Southampton, Southampton, UK; 9 National Institute for Health and Care Research Southampton Biomedical Research Centre, University Hospital Southampton, Southampton, UK

**Keywords:** Diabetes mellitus, Glycated hemoglobin A1c, Hyperglycemia, Insulin resistance, Lung cancer

## Abstract

**Background:**

The effects of glycemic status and insulin resistance on lung cancer remain unclear. We investigated the associations between both glycemic status and insulin resistance, and lung cancer mortality, in a young and middle-aged population with and without diabetes.

**Methods:**

This cohort study involved individuals who participated in routine health examinations. Lung cancer mortality was identified using national death records. Cox proportional hazards models were used to calculate hazard ratios (HRs) with 95% CIs for lung cancer mortality risk.

**Results:**

Among 666,888 individuals (mean age 39.9 ± 10.9 years) followed for 8.3 years (interquartile range, 4.6–12.7), 602 lung cancer deaths occurred. Among individuals without diabetes, the multivariable-adjusted HRs (95% CI) for lung cancer mortality comparing hemoglobin A1c categories (5.7–5.9, 6.0–6.4, and ≥ 6.5% or 39–41, 42–46, and ≥ 48 mmol/mol, respectively) with the reference (< 5.7% or < 39 mmol/mol) were 1.39 (1.13–1.71), 1.72 (1.33–2.20), and 2.22 (1.56–3.17), respectively. Lung cancer mortality was associated with fasting blood glucose categories in a dose–response manner (P for trend = 0.001) and with previously diagnosed diabetes. Insulin resistance (HOMA-IR ≥ 2.5) in individuals without diabetes was also associated with lung cancer mortality (multivariable-adjusted HR, 1.41; 95% CI, 1.13–1.75). These associations remained after adjusting for changing status in glucose, hemoglobin A1c, insulin resistance, smoking status, and other confounders during follow-up as time-varying covariates.

**Conclusions:**

Glycemic status within both diabetes and prediabetes ranges and insulin resistance were independently associated with an increased risk of lung cancer mortality.

**Supplementary Information:**

The online version contains supplementary material available at 10.1186/s40170-024-00344-4.

## Background

Lung cancer is the first and second most common cancer worldwide in men and women respectively [1], and the leading cause of cancer death despite advancements in screening and treatment [1]. Cigarette smoking remains the leading risk factor [1], though a significant proportion of lung cancer cases occur in never-smokers [2], with a recent rise in non-smoking cases [3, 4]. Additionally, a higher incidence of lung cancer among young women than men has been reported, unexplained by smoking differences [5]. Thus, identifying additional modifiable risk factors in a cohort including young and middle-aged participants may improve screening and prevention strategies, ultimately reducing lung cancer mortality.

The prevalence of diabetes is increasing worldwide, with an estimated 1 in 11 adults affected [6]. Diabetes is associated with an increased risk of cardiovascular diseases, as well as certain cancers, particularly pancreatic and liver cancers [6], and premature mortality [7]. Insulin resistance, hyperinsulinemia, and hyperglycemia, associated with diabetes, may promote cancer cell growth [8], yet studies on diabetes and lung cancer risk have shown inconsistent findings [9], ranging from positive [10, 11], to negative [12], or null [13] associations. Many studies have defined prevalent diabetes as the exposure of interest, although a few studies considered incident diabetes or time-varying diabetes status during follow-up [10, 11]. Indeed, prevalent diabetes can vary in duration, exposure to glucose-lowering medications (including insulin), and complications, making it difficult to determine the role of hyperglycemia or insulin resistance per se in lung cancer development. Furthermore, a significant proportion of individuals who have prediabetes were included in control groups although prediabetes is a state that is often accompanied by insulin resistance and is associated with an increased risk of some cancers, and also all-cause and cancer mortality [14, 15].

Currently, no single study has tested the effect of prediabetes, hemoglobin A1c (HbA1c), and measures of insulin resistance on lung cancer mortality. A Japanese study reported that elevated 2-h postload glucose levels were associated with lung cancer deaths, but did not find increased risk with elevated fasting blood glucose (FBG) levels, and did not evaluate HbA1c [16]. HbA1c is reflective of glucose concentrations in the previous 2–3 months and is more strongly associated with cardiovascular disease risk and death than fasting blood glucose (FBG) levels even in people without diabetes [17, 18]; however, no studies to date have addressed the association between HbA1c with lung cancer mortality. Insulin resistance, a key pathogenic component of diabetes, precedes diabetes [19] and may improve along with hyperglycemia through health behavior modifications before the onset of diabetes. Therefore, elucidating the association between insulin resistance and hyperglycemia and lung cancer mortality has clinical significance in the establishment of preventive measures for metabolically-associated neoplasms. Moreover, due to the high lung cancer mortality rates [1], mortality can serve as a proxy marker for incidence, and is linked to survival [20].

Hence, we investigated the associations between glycemic status in both the prediabetes and diabetes ranges, and insulin resistance, with lung cancer mortality, using a large sample of mostly young and middle-aged Korean men and women with and without diabetes.

## Methods

### Study population

This cohort study was part of the Kangbuk Samsung Health Study, which included adults who participated in health examinations at the Kangbuk Samsung Hospital Total Healthcare Centers in Seoul and Suwon, South Korea [21]. More than 80% of the participants were employees of companies and governmental organizations or their spouses, whereas the remainder voluntarily enrolled in the health examination program. In South Korea, the Industrial Safety and Health Law requires employees to undergo annual or biennial health examinations.

This study included participants who underwent health examinations between 2005 and 2019 (*n* = 682,030). A total of 15,142 participants were excluded because of unknown data on vital status (*n* = 3); missing data on FBG, HbA1c, and BMI (*n* = 1,423); and previous history of cancer (*n* = 13,734). Some participants met more than one exclusion criteria; hence, 666,888 participants were ultimately included in the analysis. This study was conducted in accordance with the Declaration of Helsinki and approved by the Institutional Review Board of Kangbuk Samsung Hospital (IRB no. KBSMC 2022–05-009), which waived the requirement for informed consent because we used a preexisting de-identified dataset of routinely collected data linked to mortality data from the Korean National Statistical Office.

### Data collection

Information on demographic characteristics, health behaviors, and medical history were collected using standardized self-administered questionnaires at baseline [21].

Smoking status was classified as never, former, or current smoker. Alcohol intake was categorized as none, < 20 g/day, or ≥ 20 g/day. Regular exercise was assessed as a weekly frequency of moderate-to-vigorous activity and categorized as < 3 and ≥ 3 times/week. Participants were considered to have a family history of cancer if ≥ 1 first-degree relative with any cancer type was present.

Trained nurses measured the sitting blood pressure, height, and weight of each participant. Hypertension was defined as blood pressure ≥ 140/90 mmHg or self-reported antihypertensive medication use. Obesity was defined as BMI ≥ 25 kg/m^2^, the proposed cutoff for diagnosing obesity in Asians [22].

Study participants were instructed to fast for at least 10 h before the blood tests, which included glycemic status markers (FBG, HbA1c, insulin); lipid levels (total cholesterol, triglycerides, high-density lipoprotein cholesterol, low-density lipoprotein cholesterol); and alanine aminotransferase and high-sensitivity C-reactive protein levels. Fasting insulin was measured using immunoradiometric assays (BioSource, Nivelles, Belgium) from 2002 to 2009, and the Modular E170 system (Roche Diagnostics, Tokyo, Japan) thereafter. Insulin resistance was assessed using the homeostatic model assessment of insulin resistance (HOMA-IR) equation with a cutoff value of 2.5 as follows: fasting blood insulin (IU/L) × FBG (mg/dL)/405 [23].

FBG levels were categorized as FBG < 90, 90–99, 100–125, and ≥ 126 mg/dL (< 5.0, 5.0–5.5, 5.6–6.9, and ≥ 7.0 mmol/L, respectively). HbA1c was categorized as < 5.7, 5.7–5.9, 6.0–6.4, and ≥ 6.5% (< 39, 39–41, 42–46, and ≥ 48 mmol/mol, respectively). Diabetes was categorized into two groups: previously diagnosed diabetes, which was defined by self-reported physician-diagnosed diabetes or current glucose-lowering medication use, and screen-detected diabetes, defined as FBG ≥ 126 mg/dL (7.0 mmol/L) or HbA1c ≥ 6.5% (48 mmol/mol) measured during health examinations.

Mortality follow-up until December 31st, 2020 was based on nationwide death certificate data retrieved from the Korean National Statistical Office, which provided the date and cause of death according to the International Statistical Classification of Diseases and Related Health Problems, Tenth Revision (ICD-10). Death certificate data are virtually 100% complete because of the legal requirement to report deaths in Korea. The majority (94.9%) of people with cancer of any site as the cause of death were also found to have a record of cancer diagnosis in the medical utilization data [24]. Lung cancer mortality was defined as death due to malignant neoplasm of the trachea, bronchus, and lung (C33 and C34 in the ICD-10).

### Statistical analysis

We used descriptive statistics to analyze participants’ characteristics according to lung cancer mortality. Owing to differences in age and sex among patients with and without lung cancer mortality, the baseline characteristics are shown as age- and sex-adjusted means or proportions and 95% CIs.

The primary outcome was lung cancer mortality. Patients who died owing to other causes were censored at the date of death. We also performed sensitivity analyses to minimize subclinical cancer events at baseline by excluding lung cancer mortality within the first 2–4 years of follow-up.

Cox proportional hazards regression analyses were performed to compute hazard ratios (HRs) and 95% CIs for lung cancer mortality. Age was used as the timescale; we considered the age when participants received their first health examination (left truncation) and when they exited the analysis, on the date of their death or on December 31, 2020. This approach effectively controlled for age. The proportional hazards assumption was assessed by analyzing graphs of the estimated log (-log[SURVIVAL]); no violation of the assumption was found.

Models analyzing the association between glycemic status and lung cancer mortality were initially adjusted for age (as the timescale) and sex. Models were then additionally adjusted for potential confounders. These included study center (Seoul or Suwon); screening year; smoking status (never, ever, current smoker, or unknown); regular exercise (< 3, ≥ 3 times/week, or unknown); BMI; education level (< community college graduate, ≥ community college graduate, or unknown); history of hypertension; dyslipidemia medication use; history of chronic obstructive pulmonary disease (COPD); history of asthma; and family history of cancer. Additionally, to evaluate the impact of changes in glycemic status markers and other covariates and potential confounders, between baseline and follow-up, we introduced FBG, HbA1c, HOMA-IR, smoking status, and other potential confounders as time-varying covariates in additional analyses. To test for linear trends, the median value for each category was included as a continuous variable in each model.

Subgroup analyses were performed according to age (< 50 and ≥ 50 years), sex (female and male), and smoking status (never or ever). Interactions between glycemic status categories and subgroup characteristics were tested using likelihood ratio tests to compare models with and without multiplicative interaction terms. We additionally examined the association between diabetes duration and age at diagnosis of diabetes with lung cancer mortality. We also examined the association between waist circumference and lung cancer mortality, as waist circumference is associated with insulin resistance [25].

Statistical analyses were performed using Stata version 17.0 (StataCorp LP, College Station, TX, USA). The reported *p* values were two-tailed, and *p* values < 0.05 indicated statistical significance.

## Results

The baseline participant characteristics are shown in Table 1. The mean age of participants was 39.9 years (SD, 10.9; median, 37; interquartile range, 31.6–45.4 years). The mean age of participants who died of lung cancer was 59.3 years at baseline, in contrast to 39.9 years in those who did not die of lung cancer during follow-up. Lung cancer mortality was positively associated with older age, male sex, current smoking status, COPD, and asthma and inversely associated with BMI, education level, dyslipidemia medication use, and family history of cancer.

**Table 1:** Estimated^a^ mean values (95% CI) and adjusted^a^ proportions (95% CI) of baseline characteristics of study participants according to lung cancer mortality (*n* = 666,888)

Characteristics	Lung cancer mortality (-)	Lung cancer mortality ( +)	*p-*value
Number	666,286	602	
Age (years)	39.9 (39.9–40.0)	59.3 (58.4–60.2)	< 0.001
Male sex (%)	52.3 (52.2–52.5)	79.0 (75.7–82.3)	< 0.001
BMI (kg/m^2^)	23.4 (23.4–23.4)	22.0 (21.7–22.2)	< 0.001
Obesity (%)^b^	29.0 (28.9–29.1)	18.6 (16.1–21.1)	< 0.001
Current smoker (%)	21.8 (21.7–21.9)	35.6 (32.7–38.5)	< 0.001
Alcohol intake (%)^c^	19.4 (19.3–19.5)	22.4 (19.5–25.3)	0.033
Regular exercise (%)^d^	15.1 (15.0–15.1)	12.9 (10.6–15.2)	0.084
High education level (%)^e^	75.8 (75.7–75.9)	61.1 (56.4–65.7)	< 0.001
Hypertension (%)	14.0 (13.9–14.0)	9.9 (8.5–11.3)	< 0.001
Diabetes (%)	4.7 (4.7–4.8)	4.5 (3.7–5.3)	0.525
Glucose-lowering medication (%)	2.1 (2.1–2.1)	1.5 (1.1–1.9)	0.015
Lipid-lowering medication (%)	2.9 (2.9–3.0)	0.9 (0.5–1.3)	< 0.001
Family history of cancer (%)	24.1 (24.0–24.2)	17.6 (15.1–20.2)	< 0.001
History of CVD (%)	3.4 (3.3–3.4)	4.3 (3.3–5.2)	0.042
History of COPD (%)	18.4 (18.3–18.5)	31.3 (27.7–34.9)	< 0.001
History of asthma (%)	1.5 (1.4–1.5)	2.5 (1.4–3.6)	0.025
Systolic BP (mmHg)	111.4 (111.3–111.4)	111.2 (110.2–112.2)	0.697
Diastolic BP (mmHg)	71.4 (71.4–71.5)	70.0 (69.2–70.7)	< 0.001
Glucose (mg/dL)	95.3 (95.3–95.3)	96.8 (95.5–98.0)	0.018
HbA1c (%)	5.5 (5.5–5.5)	5.6 (5.6–5.6)	0.009
Total cholesterol (mg/dL)	191.6 (191.5–191.7)	186.2 (183.5–188.9)	< 0.001
LDL-C (mg/dL)	117.3 (117.2–117.4)	104.5 (102.0–107.0)	< 0.001
HDL-C (mg/dL)	58.0 (57.9–58.0)	58.2 (57.1–59.3)	0.631
Triglycerides (mg/dL)	114.3 (114.1–114.5)	118.2 (112.1–124.2)	0.443
ALT (U/L)	24.7 (24.6–24.8)	21.8 (20.0–23.6)	< 0.001
HOMA-IR^f^	1.67 (1.67–1.68)	1.72 (1.62–1.82)	0.570
Insulin (μIU/mL)^f^	7.17 (7.16–7.18)	7.42 (7.01–7.82)	0.378

*ALT* alanine aminotransferase, *BMI* body mass index, *BP* blood pressure, *CVD* cardiovascular disease, *COPD* chronic obstructive pulmonary disease, *HbA1c* hemoglobin A1c, *HDL-C* high-density lipoprotein cholesterol, *HOMA-IR* homeostasis model assessment of insulin resistance, *LDL-C* low-density lipoprotein cholesterol

^a^Adjusted for age and sex

^b^BMI ≥ 25 kg/m^2^

^c^ ≥ 20 g/day

^d^ ≥ 3 times/week

^e^ ≥ college graduate

^f^among 658,380 subjects with available insulin data

During 5,681,158 person-years of follow-up, 602 incident lung cancer deaths occurred, and the total lung cancer mortality rate was 10.6 per 10^5^ person-years. The median follow-up duration was 8.3 years (interquartile range, 4.6–12.7; maximum, 15.8 years). After adjustment for age and sex, increased HbA1c and FBG levels and previously diagnosed diabetes were positively associated with lung cancer mortality (Table 2). After adjusting for additional confounders, the associations of increased FBG and HbA1c levels and previously diagnosed diabetes with lung cancer mortality were consistently observed. The multivariable-adjusted HRs (95% CIs) for lung cancer mortality comparing HbA1c categories (5.7–5.9, 6.0–6.4, ≥ 6.5%) and previously diagnosed diabetes with HbA1c < 5.7% as the reference are shown in Table 2.

**Table 2:** Hazard ratios (95% CIs) for lung cancer mortality by glycemic status (*n* = 666,888)

	**Person-years**	**Number of events**	**Mortality rate (10**^**5**^** PY)**	**Age- and sex-adjusted HR (95% CI)**	**Multivariable-adjusted HR**^**a**^** (95% CI)**	**HR (95% CI)**^**b**^** per the model using time-dependent variables**
**HbA1c category**^**c**^** (%)**						
< 5.7	4,119,484	249	6.0	1.00 (reference)	1.00 (reference)	1.00 (reference)
5.7–5.9	1,027,740	148	14.4	1.31 (1.07–1.62)	1.39 (1.13–1.71)	1.28 (1.03–1.57)
6.0–6.4	303,919	92	30.3	1.58 (1.23–2.01)	1.72 (1.33–2.20)	1.62 (1.26–2.08)
≥ 6.5 (screen-detected diabetes)	78,782	37	47.0	2.00 (1.41–2.83)	2.22 (1.56–3.17)	2.02 (1.39–2.93)
P for trend				< 0.001	< 0.001	< 0.001
Previously diagnosed diabetes	151,233	76	50.3	1.35 (1.04–1.76)	1.54 (1.18–2.03)	1.52 (1.17–1.96)
**FBG category**^**d**^** (mg/dL)**						
< 90	1,916,655	110	5.7	1.00 (reference)	1.00 (reference)	1.00 (reference)
90–99	2,428,299	190	7.8	0.97 (0.76–1.22)	1.05 (0.83–1.33)	1.11 (0.87–1.41)
100–125	1,112,086	200	18.0	1.18 (0.93–1.49)	1.38 (1.09–1.75)	1.39 (1.09–1.78)
≥ 126 (screen-detected diabetes)	72,886	26	35.7	1.45 (0.94–2.22)	1.71 (1.11–2.63)	1.63 (1.04–2.56)
P for trend				0.048	0.001	0.001
Previously diagnosed diabetes	151,233	76	50.3	1.18 (0.87–1.59)	1.43 (1.05–1.94)	1.49 (1.11–2.01)
**Insulin Resistance**^**e**^						
HOMA-IR < 2.5	4,453,269	380	8.5	1.00 (reference)	1.00 (reference)	1.00 (reference)
HOMA-IR ≥ 2.5	1,018,854	123	12.1	1.15 (0.94–1.41)	1.41 (1.13–1.75)	1.28 (1.01–1.61)

*BMI* body mass index, *CI* confidence interval, *COPD* chronic obstructive pulmonary disease, *FBG* fasting blood glucose, *HbA1c* hemoglobin A1c, *HR* hazard ratio, *HOMA-IR* homeostasis model assessment of insulin resistance, *PY* person-years

^a^Cox proportional hazard models were used with age as a timescale to estimate HRs and 95% CIs. The multivariable model was adjusted for age (timescale), sex, center, screening year, smoking status, regular exercise, BMI, education level, history of hypertension, dyslipidemia medication use, history of COPD, history of asthma, and family history of cancer

^b^Estimated from Cox proportional hazard models with FBG, HbA1c, and HOMA-IR categories; smoking status; exercise; BMI; dyslipidemia medication use; and history of hypertension, COPD, and asthma as time-dependent categorical variables and baseline age, sex, center, examination year, education level, and family history of cancer as time-fixed variables

^c^HbA1c < 5.7, 5.7–5.9, 6.0–6.4, and ≥ 6.5% corresponds to < 39, 39–41, 42–46, and ≥ 48 mmol/mol, respectively

^d^FBG < 90, 90–99, 100–125, and ≥ 126 mg/dL corresponds to < 5.0, 5.0–5.5, 5.6–6.9, and ≥ 7.0 mmol/L, respectively

^e^Among subjects without previously diagnosed diabetes

Insulin resistance defined as HOMA-IR ≥ 2.5 among participants without diabetes also showed a positive association with lung cancer mortality after adjustments for confounders, with a multivariable-adjusted HR (95% CI) of 1.41 (1.13–1.75). The observed associations remained significant in the time-dependent models including glycemic status markers (FBG, HbA1c), insulin resistance, and confounders analyzed as time-varying covariates (Table 2).

In the analysis according to clinically relevant subgroups, there were no significant interactions in associations of HbA1c concentration, FBG level, and insulin resistance with lung cancer mortality (Supplementary Tables S1 and S2).

In the sensitivity analysis excluding lung cancer mortality during the first 2, 3, and 4 years of follow-up, the associations of glycemic status, insulin resistance, and diabetes with lung cancer mortality remained significant (Supplementary Table S3). There was no evidence of increased risk with increasing diabetes duration and age at diagnosis of diabetes with lung cancer mortality (Supplementary Table S4 and S5). We additionally found that abdominal obesity was associated with lung cancer mortality (multivariable-adjusted HR 1.72, 95% CI 1.34–2.22), and that each 1-cm increase in waist circumference was also associated with lung cancer mortality (multivariable-adjusted HR 1.05, 95% CI 1.02–1.07) (Supplementary Table S6).

## Discussion

In this retrospective cohort study, hyperglycemia based on both FBG and HbA1c levels was associated with lung cancer mortality risk in a dose–response manner among participants without previously diagnosed diabetes, and the increased risk began within the prediabetes range. Both screen-detected and previously diagnosed diabetes and insulin resistance were independently associated with lung cancer mortality. Our study included the analysis of multiple major confounders and health behaviors, and adjustments for these factors did not materially affect the associations. These associations also remained consistent when temporal changes in glycemic status, insulin resistance, and health behaviors over time during follow-up were treated as time-varying covariates. Moreover, there were no significant interactions among the subgroups, and notably, the association between insulin resistance and lung cancer mortality persisted in never-smokers. Our results suggest that hyperglycemia and insulin resistance are independently associated with lung cancer mortality, even in individuals without diabetes.

Previous studies analyzing the relationship between hyperglycemia in the prediabetes range and lung cancer are scarce [16]. A Japanese cohort study reported that impaired glucose tolerance assessed by the 2-h post-load glucose concentration in the non-diabetes range was associated with lung cancer mortality, but did not adjust for important confounders such as smoking status, and reported a null association for impaired fasting hyperglycemia [16]. Another cohort study reported that increased HbA1c was associated with increased lung cancer risk, although it included diabetes in the analysis by adjusting for it as a confounding variable and did not analyze associations in the prediabetes range [26]. Our study is the first to show that both increased FBG and HbA1c levels in the prediabetes range are associated with lung cancer mortality among individuals without diabetes.

Furthermore, our study is the first to include a cohort of both men and women and show an independent association between insulin resistance and lung cancer mortality among individuals without diabetes. Waist circumference, which is associated with insulin resistance [25], was also associated with lung cancer mortality, consistent with a previous study which showed increased risk of lung cancer mortality with higher waist circumference [27]. A few studies have suggested that insulin resistance is associated with lung cancer risk [28–30], yet others failed to show an association with lung cancer mortality [31]. One study reported a positive association between HOMA-IR and lung cancer risk; however, this was a cross-sectional study that did not assess diabetes status [28].

A cohort study reported an increased lung cancer risk in the highest quartile of insulin levels with a similar trend among people without diabetes; however the study included only male smokers and the association was not found in people within higher glucose quartiles [29]. Similarly, a Mendelian randomization study suggested an association between fasting insulin and lung cancer risk [30], whereas another study reported no association between type 2 diabetes and lung cancer risk [32]. In light of these findings, insulin resistance with hyperinsulinemia may be the key factor influencing the association between glycemic status and lung cancer risk. One study that analyzed cancer incidence according to diabetes duration found that cancer incidence peaked along with C-peptide levels at 4–8 years after diabetes diagnosis, whereas with longer durations of diabetes, cancer risk gradually decreased along with C-peptide levels, probably owing to β-cell exhaustion, indicating a role of hyperinsulinemia in cancer development [11]. Insulin resistance plays an important role in the pathogenesis of type 2 diabetes [19]. In addition, glucose-lowering medications show inconsistent associations with lung cancer risk, that is, an apparent decreased risk with metformin [33] and increased risk with insulin and insulin secretagogues, with potential for confounding by indication [34].

Regarding the association between diabetes and lung cancer risk, previous cohort studies have reported inconsistent findings [10, 12], and meta-analyses have reported no significant associations overall in men and increased risk among women [9, 35]. The substantial heterogeneity between the included studies may be responsible for these inconsistencies. The heterogeneity between studies in men was substantial, whereas the heterogeneity between studies in women was low [9, 35]. In our study, there was no significant interaction by sex for the association between glycemic status and insulin resistance and lung cancer mortality, although there were a smaller number of lung cancer deaths in women (123 vs. 479). While previously diagnosed diabetes was associated with increased lung cancer mortality risk, the risk was even stronger in screen-detected diabetes. Other studies have also suggested that cancer risk is highest in the years immediately after diabetes diagnosis, with the potential for reverse causality [10]. Nonetheless, the association between glycemic status, insulin resistance, and lung cancer mortality remained consistent in our sensitivity analysis in which we excluded cancer events during the first 2 to 4 years of follow-up.

Lung cancer is generally diagnosed among older populations; according to a national lung cancer patient registry in Korea, the median age at diagnosis was 70 years [36]. Although we did not have data on lung cancer incidence, the median age at death among participants who died of lung cancer was even lower at 68 years (interquartile range, 60.1 to 74.5 years), probably because our cohort consisted of mostly a young and middle-aged population. Previous studies analyzing young vs. older lung cancer patients reported that younger patients had more advanced stage at diagnosis [37, 38]. The observed higher lung cancer mortality in a relatively young population with hyperglycemia and insulin resistance might be attributed to these later-stage diagnoses. However, further research is necessary, as specific information on cancer stages and incidence rates was not available for this study. Nonetheless, the associations between glucose levels, HbA1c, and insulin resistance with lung cancer mortality did not differ in the subgroup analysis of the age groups < 50 vs. ≥ 50.

Several plausible mechanisms may underlie the association between insulin resistance, glycemic status, and lung cancer mortality. Increased insulin exposure due to insulin resistance or even insulin administration in patients with diabetes may contribute to carcinogenesis [8] through the upregulation of IGF-I activity or downregulation of IGF-binding protein-1 activity [39]. IGF-I is involved in cell proliferation, migration, and apoptosis [40], and IGF-I and IGF-II levels were reportedly found in higher levels in bronchial tissue with high-grade dysplasia than in normal tissue [41]. Insulin also stimulates the Ras signaling pathway, which is important in lung carcinogenesis [42] and may also stimulate local angiogenesis [43] or promote tumor cell growth through insulin receptors on lung cancer cells [44]. In turn, hyperglycemia causes oxidative stress and chronic inflammation, which may cause damage to DNA and the lungs [45], increasing susceptibility to carcinogenesis and promoting cancer proliferation through the induction of epidermal growth factor. Hyperglycemia may also promote tumor invasion and metastasis through upregulation of the transforming growth factor-beta1/phosphoinositide 3-kinase/protein kinase B signaling pathway [46].

### Strengths and limitations

Our study included a large longitudinal cohort of mostly young and middle-aged participants free of cancer at baseline, with a long follow-up period and adjustments for multiple confounders. However, our study had some limitations. First, information on the type of diabetes and glucose-lowering medication was unavailable. However, most people in our cohort had type 2 diabetes [6], and the prevalence of type 1 diabetes is reported as only 1.19% of all patients living with diabetes in Korea [47]. In addition, in our study, all previously diagnosed and screen-detected diabetes and insulin resistance, a pathogenic factor for type 2 diabetes, was associated with lung cancer mortality. Second, screen-detected diabetes was identified using a single FBG and HbA1c measurement, whereas guidelines recommend repeated testing for confirmation of diabetes diagnosis. However, HbA1c levels reflect chronic hyperglycemia and exhibit low biological variability [18]. In addition, our main results remained unchanged in participants with screen-detected diabetes identified through both FBG and HbA1c levels, in previously diagnosed diabetes, and in analyses using time-varying covariates that accounted for any change in glucose status between baseline and follow-up. Third, insulin resistance was based on HOMA-IR and not on euglycemic insulin clamp analysis, which is the gold standard method for the assessment of insulin sensitivity. However, HOMA-IR correlates well with hyperinsulinemic–euglycemic insulin clamp data assessing whole-body insulin sensitivity [48], and insulin clamps are impractical in routine health examinations. Fourth, information on lung cancer histology and incident lung cancer was not available. While the association between glycemic status and insulin resistance with lung cancer mortality may differ by the histologic subtype, a previous study reported that the association between insulin levels and lung cancer risk did not differ across histological subtypes [29]. However, other studies reported an association between higher glycemic index and squamous cell carcinoma (SCC) [49], and elevated GLUT1 expression in SCC [50] compared to adenocarcinoma, warranting further studies analyzing the association between glycemic status and lung cancer mortality by histopathology. Regarding lack of lung cancer incidence, because of its short median survival, lung cancer incidence approximates its high mortality [1]. In Korea, the 5-year relative survival rate for lung cancer was low (16.5% in 2001–2005 and 30.2% in 2013–2017) [51]. Hence, similar findings for lung cancer incidence may be expected, although it is not possible to separate the effects of glycemic status on incidence and survival from studies on cancer mortality. The association between diabetes and poor lung cancer prognosis [52] may also have contributed to the associations observed in our study. Lung cancer mortality is likely to represent lung cancer with a poor prognosis; hence we may have missed lung cancer with a more indolent course. Nonetheless, people who developed incident lung cancer during follow-up but did not die would have been categorized into the control group, creating a misclassification bias that would attenuate the strength of the associations observed in our study towards the null. Fifth, although our cohort included available data for multiple confounders, data on risk factors such as occupational exposure and passive smoking were unavailable, and unmeasured or residual confounders could not be excluded. Finally, because our study included young and middle-aged Koreans, the results may not be generalizable to populations with different characteristics.

## Conclusions

In conclusion, hyperglycemia and insulin resistance among individuals without diabetes, as well as individuals with previously diagnosed and screen-detected diabetes, were associated with an increased risk of lung cancer mortality, regardless of smoking status. Further studies are required to investigate whether theses associations exist for lung cancer incidence, and whether interventions to treat hyperglycemia and insulin resistance, such as increased physical activity, weight loss, and healthy dietary habits, reduce the incidence and mortality of lung cancer.

### Supplementary Information

Supplementary Material 1: Supplementary Table S1. Hazard ratios (95% CIs) for lung cancer mortality per glucose and HbA1c category in clinically relevant subgroups. Supplementary Table S2. Hazard ratios (95% CIs) for lung cancer mortality by insulin resistance in clinically relevant subgroups. Supplementary Table S3. Hazard ratios (95% CIs) for lung cancer mortality per glucose category in the overall population after excluding lung cancer mortality cases that occurred during the first 2–4 years of the follow-up period. Supplementary Table S4. Hazard ratios (95% CIs) for lung cancer mortality by glycemic status and duration of diabetes (*n* = 658,973). Supplementary Table S5. Hazard ratios (95% CIs) for lung cancer mortality by glycemic status and age at diabetes diagnosis (*n* = 658,973). Supplementary Table S6. Hazard ratios (95% CIs) for lung cancer mortality by waist circumference (*n* = 562,111).

## Data Availability

The data underlying this article are not available for public distribution as we do not have permission from the IRB. Supporting information or data is available from the corresponding author upon reasonable request.

## Abbreviations

BMI: Body mass index
CI: Confidence interval
COPD: Chronic obstructive pulmonary disease
FBG: Fasting blood glucose
HbA1c: Hemoglobin A1c
HOMA-IR: Homeostasis model assessment of insulin resistance
HR: Hazard ratio

## References

[1] Sung H, Ferlay J, Siegel RL, Laversanne M, Soerjomataram I, Jemal A (2021). Global Cancer Statistics 2020: GLOBOCAN Estimates of Incidence and Mortality Worldwide for 36 Cancers in 185 Countries. CA Cancer J Clin.

[2] Couraud S, Zalcman G, Milleron B, Morin F, Souquet PJ (2012). Lung cancer in never smokers–a review. Eur J Cancer.

[3] Pelosof L, Ahn C, Gao A, Horn L, Madrigales A, Cox J (2017). Proportion of Never-Smoker Non-Small Cell Lung Cancer Patients at Three Diverse Institutions. J Natl Cancer Inst..

[4] Cufari ME, Proli C, De Sousa P, Raubenheimer H, Al Sahaf M, Chavan H (2017). Increasing frequency of non-smoking lung cancer: Presentation of patients with early disease to a tertiary institution in the UK. Eur J Cancer.

[5] Jemal A, Miller KD, Ma J, Siegel RL, Fedewa SA, Islami F (2018). Higher Lung Cancer Incidence in Young Women Than Young Men in the United States. N Engl J Med.

[6] Zheng Y, Ley SH, Hu FB (2018). Global aetiology and epidemiology of type 2 diabetes mellitus and its complications. Nat Rev Endocrinol.

[7] Rao Kondapally Seshasai S, Kaptoge S, Thompson A, Di Angelantonio E, Gao P, Sarwar N (2011). Diabetes mellitus, fasting glucose, and risk of cause-specific death. N Engl J Med.

[8] Giovannucci E, Harlan DM, Archer MC, Bergenstal RM, Gapstur SM, Habel LA (2010). Diabetes and cancer: a consensus report. Diabetes Care.

[9] Yi ZH, Luther Y, Xiong GH, Ni YL, Yun F, Chen J (2020). Association between diabetes mellitus and lung cancer: Meta-analysis. Eur J Clin Invest.

[10] Dankner R, Boffetta P, Balicer RD, Boker LK, Sadeh M, Berlin A (2016). Time-Dependent Risk of Cancer After a Diabetes Diagnosis in a Cohort of 2.3 Million Adults. Am J Epidemiol..

[11] Hu Y, Zhang X, Ma Y, Yuan C, Wang M, Wu K (2021). Incident Type 2 Diabetes Duration and Cancer Risk: A Prospective Study in Two US Cohorts. J Natl Cancer Inst.

[12] Atchison EA, Gridley G, Carreon JD, Leitzmann MF, McGlynn KA (2011). Risk of cancer in a large cohort of U.S. veterans with diabetes. Int J Cancer..

[13] Hall GC, Roberts CM, Boulis M, Mo J, MacRae KD (2005). Diabetes and the risk of lung cancer. Diabetes Care.

[14] Huang Y, Cai X, Qiu M, Chen P, Tang H, Hu Y (2014). Prediabetes and the risk of cancer: a meta-analysis. Diabetologia.

[15] Cai X, Zhang Y, Li M, Wu JH, Mai L, Li J (2020). Association between prediabetes and risk of all cause mortality and cardiovascular disease: updated meta-analysis. BMJ.

[16] Hirakawa Y, Ninomiya T, Mukai N, Doi Y, Hata J, Fukuhara M (2012). Association between glucose tolerance level and cancer death in a general Japanese population: the Hisayama Study. Am J Epidemiol..

[17] Selvin E, Steffes MW, Zhu H, Matsushita K, Wagenknecht L, Pankow J (2010). Glycated hemoglobin, diabetes, and cardiovascular risk in nondiabetic adults. N Engl J Med.

[18] Sacks DB (2011). A1C versus glucose testing: a comparison. Diabetes Care.

[19] Fujimoto WY (2000). The importance of insulin resistance in the pathogenesis of type 2 diabetes mellitus. Am J Med.

[20] Ellis L, Woods LM, Esteve J, Eloranta S, Coleman MP, Rachet B (2014). Cancer incidence, survival and mortality: explaining the concepts. Int J Cancer.

[21] Chang Y, Ryu S, Choi Y, Zhang Y, Cho J, Kwon MJ (2016). Metabolically Healthy Obesity and Development of Chronic Kidney Disease: A Cohort Study. Ann Intern Med.

[22] World Health Organization. The Asia-Pacific Perspective : Redefining Obesity and Its Treatment. Sydney, Austrailia: Health Communications Australia Pty Limit; 2000. Available from: https://iris.who.int/handle/10665/206936.

[23] Matthews DR, Hosker JP, Rudenski AS, Naylor BA, Treacher DF, Turner RC (1985). Homeostasis model assessment: insulin resistance and beta-cell function from fasting plasma glucose and insulin concentrations in man. Diabetologia.

[24] Song YM, Sung J (2001). Body mass index and mortality: a twelve-year prospective study in Korea. Epidemiology.

[25] Ramirez-Manent JI, Jover AM, Martinez CS, Tomas-Gil P, Marti-Lliteras P, Lopez-Gonzalez AA (2023). Waist Circumference Is an Essential Factor in Predicting Insulin Resistance and Early Detection of Metabolic Syndrome in Adults. Nutrients..

[26] Srour B, Kaaks R, Johnson T, Hynes LC, Kuhn T, Katzke VA (2022). Ageing-related markers and risks of cancer and cardiovascular disease: a prospective study in the EPIC-Heidelberg cohort. Eur J Epidemiol.

[27] Leitzmann MF, Moore SC, Koster A, Harris TB, Park Y, Hollenbeck A (2011). Waist circumference as compared with body-mass index in predicting mortality from specific causes. PLoS ONE.

[28] Petridou ET, Sergentanis TN, Antonopoulos CN, Dessypris N, Matsoukis IL, Aronis K (2011). Insulin resistance: an independent risk factor for lung cancer?. Metabolism.

[29] Argirion I, Weinstein SJ, Mannisto S, Albanes D, Mondul AM (2017). Serum Insulin, Glucose, Indices of Insulin Resistance, and Risk of Lung Cancer. Cancer Epidemiol Biomarkers Prev.

[30] Carreras-Torres R, Johansson M, Haycock PC, Wade KH, Relton CL, Martin RM (2017). Obesity, metabolic factors and risk of different histological types of lung cancer: A Mendelian randomization study. PLoS ONE.

[31] Parekh N, Lin Y, Hayes RB, Albu JB, Lu-Yao GL (2010). Longitudinal associations of blood markers of insulin and glucose metabolism and cancer mortality in the third National Health and Nutrition Examination Survey. Cancer Causes Control.

[32] Hong T, Qin N, Zhao X, Wang C, Jiang Y, Ma H (2021). Investigation of Causal Effect of Type 2 Diabetes Mellitus on Lung Cancer: A Mendelian Randomization Study. Front Genet.

[33] Currie CJ, Poole CD, Jenkins-Jones S, Gale EA, Johnson JA, Morgan CL (2012). Mortality after incident cancer in people with and without type 2 diabetes: impact of metformin on survival. Diabetes Care.

[34] Chang CH, Lin JW, Wu LC, Lai MS, Chuang LM (2012). Oral insulin secretagogues, insulin, and cancer risk in type 2 diabetes mellitus. J Clin Endocrinol Metab.

[35] Lee JY, Jeon I, Lee JM, Yoon JM, Park SM (2013). Diabetes mellitus as an independent risk factor for lung cancer: a meta-analysis of observational studies. Eur J Cancer.

[36] Choi CM, Kim HC, Jung CY, Cho DG, Jeon JH, Lee JE (2019). Report of the Korean Association of Lung Cancer Registry (KALC-R), 2014. Cancer Res Treat.

[37] Liam CK, Lim KH, Wong CM (2000). Lung cancer in patients younger than 40 years in a multiracial Asian country. Respirology.

[38] Skarin AT, Herbst RS, Leong TL, Bailey A, Sugarbaker D (2001). Lung cancer in patients under age 40. Lung Cancer.

[39] Snyder DK, Clemmons DR (1990). Insulin-dependent regulation of insulin-like growth factor-binding protein-1. J Clin Endocrinol Metab.

[40] Denley A, Cosgrove LJ, Booker GW, Wallace JC, Forbes BE (2005). Molecular interactions of the IGF system. Cytokine Growth Factor Rev.

[41] Kim WY, Jin Q, Oh SH, Kim ES, Yang YJ, Lee DH (2009). Elevated epithelial insulin-like growth factor expression is a risk factor for lung cancer development. Cancer Res.

[42] Mascaux C, Iannino N, Martin B, Paesmans M, Berghmans T, Dusart M (2005). The role of RAS oncogene in survival of patients with lung cancer: a systematic review of the literature with meta-analysis. Br J Cancer.

[43] Rensing KL, Houttuijn Bloemendaal FM, Weijers EM, Richel DJ, Buller HR, Koolwijk P (2010). Could recombinant insulin compounds contribute to adenocarcinoma progression by stimulating local angiogenesis?. Diabetologia.

[44] Zhang H, Fagan DH, Zeng X, Freeman KT, Sachdev D, Yee D (2010). Inhibition of cancer cell proliferation and metastasis by insulin receptor downregulation. Oncogene.

[45] Lorenzi M, Montisano DF, Toledo S, Barrieux A (1986). High glucose induces DNA damage in cultured human endothelial cells. J Clin Invest.

[46] Kang X, Kong F, Wu X, Ren Y, Wu S, Wu K (2015). High glucose promotes tumor invasion and increases metastasis-associated protein expression in human lung epithelial cells by upregulating heme oxygenase-1 via reactive oxygen species or the TGF-beta1/PI3K/Akt signaling pathway. Cell Physiol Biochem.

[47] Song SO, Song YD, Nam JY, Park KH, Yoon JH, Son KM (2016). Epidemiology of Type 1 Diabetes Mellitus in Korea through an Investigation of the National Registration Project of Type 1 Diabetes for the Reimbursement of Glucometer Strips with Additional Analyses Using Claims Data. Diabetes Metab J.

[48] Katz A, Nambi SS, Mather K, Baron AD, Follmann DA, Sullivan G (2000). Quantitative insulin sensitivity check index: a simple, accurate method for assessing insulin sensitivity in humans. J Clin Endocrinol Metab.

[49] Melkonian SC, Daniel CR, Ye Y, Pierzynski JA, Roth JA, Wu X (2016). Glycemic Index, Glycemic Load, and Lung Cancer Risk in Non-Hispanic Whites. Cancer Epidemiol Biomarkers Prev.

[50] Goodwin J, Neugent ML, Lee SY, Choe JH, Choi H, Jenkins DMR (2017). The distinct metabolic phenotype of lung squamous cell carcinoma defines selective vulnerability to glycolytic inhibition. Nat Commun.

[51] Lee JG, Kim HC, Choi CM (2021). Recent Trends of Lung Cancer in Korea. Tuberc Respir Dis (Seoul).

[52] Bi G, Yao G, Bian Y, Xue L, Zhang Y, Lu T (2020). The Effect of Diabetes Mellitus on Prognosis of Patients with Non-Small-Cell Lung Cancer: A Systematic Review and Meta-Analysis. Ann Thorac Cardiovasc Surg.

