# Supplementary material for: Glycemic status, insulin resistance, and mortality from lung cancer among individuals with and without diabetes
Source: Cancer Metab. 2024 Jun 20;12:17. doi: 10.1186/s40170-024-00344-4 (PMC11188269; doi:10.1186/s40170-024-00344-4)
Supplement: Supplementary file 1 — Supplementary Material 1: Supplementary Table S1. Hazard ratios (95% CIs) for lung cancer mortality per glucose and HbA1c category in clinically relevant subgroups. Supplementary Table S2. Hazard ratios (95% CIs) for lung cancer mortality by insulin resistance in clinically relevant subgroups. Supplementary Table S3. Hazard ratios (95% CIs) for lung cancer mortality per glucose category in the overall population after excluding lung cancer mortality cases that occurred during the first 2–4 years of the follow-up period. Supplementary Table S4. Hazard ratios (95% CIs) for lung cancer mortality by glycemic status and duration of diabetes (n = 658,973). Supplementary Table S5. Hazard ratios (95% CIs) for lung cancer mortality by glycemic status and age at diabetes diagnosis (n = 658,973). Supplementary Table S6. Hazard ratios (95% CIs) for lung cancer mortality by waist circumference (n = 562,111). [file 40170_2024_344_MOESM1_ESM.docx]

**Supplementary Table S1** Hazard ratios (95% CIs) for lung cancer mortality per glucose and HbA1c category in clinically relevant subgroups

| **Subgroup** | **Glycemic status** | | | | | | ***p* for interaction** |
| --- | --- | --- | --- | --- | --- | --- | --- |
| **HbA1c category^a^ (%)** | **< 5.7** | **5.7–5.9** | **6.0–6.4** | **≥ 6.5**  **(Screen-detected diabetes)** | ***p* for trend** | **Previously diagnosed diabetes** |  |
| Age (years) |  |  |  |  |  |  | 0.117 |
| < 50 (*n* = 549,133) | Reference | 1.02 (0.64–1.62) | 1.15 (0.69–1.93) | 3.80 (1.65–8.74) | 0.025 | 2.20 (0.91–5.35) |  |
| ≥ 50 (*n* = 117,755) | Reference | 1.07 (0.81–1.40) | 1.43 (1.09–1.87) | 1.41 (0.85–2.33) | 0.010 | 1.39 (1.00–1.92) |  |
| Sex |  |  |  |  |  |  | 0.689 |
| Female (*n* = 317,664) | Reference | 0.83 (0.53–1.32) | 1.09 (0.67–1.76) | 1.15 (0.35–3.76) | 0.749 | 1.02 (0.51–2.02) |  |
| Male (*n* = 349,224) | Reference | 1.15 (0.87–1.52) | 1.50 (1.14–1.98) | 1.89 (1.18–3.02) | < 0.001 | 1.58 (1.12–2.22) |  |
| Ever smoker |  |  |  |  |  |  | 0.647 |
| No (*n* = 367,550) | Reference | 0.86 (0.55–1.34) | 1.21 (0.77–1.90) | 1.54 (0.6–3.95) | 0.434 | 0.88 (0.45–1.75) |  |
| Yes (*n* = 264,119) | Reference | 1.10 (0.82–1.47) | 1.43 (1.07–1.91) | 1.80 (1.08–3.01) | 0.001 | 1.61 (1.12–2.30) |  |
| **FBG category^b^ (mg/dl)** | **< 90** | **90–99** | **100–125** | **≥ 126**  **(Screen-detected diabetes)** | ***p* for trend** | **Previously diagnosed diabetes** | ***p* for interaction** |
| Age (years) |  |  |  |  |  |  | 0.758 |
| < 50 (*n* = 549,133) | Reference | 1.19 (0.73–1.94) | 1.68 (0.81–3.49) | 3.00 (1.21–7.46) | 0.007 | 2.20 (0.95–5.09) |  |
| ≥ 50 (*n* = 117,755) | Reference | 1.41 (1.12–1.78) | 1.70 (1.30–2.22) | 2.10 (1.44–3.09) | < 0.001 | 1.49 (1.12–1.98) |  |
| Sex |  |  |  |  |  |  | 0.136 |
| Female (*n* = 317,664) | Reference | 1.44 (0.94–2.19) | 0.96 (0.53–1.73) | 2.65 (1.29–5.47) | 0.545 | 1.30 (0.67–2.54) |  |
| Male (*n* = 349,224) | Reference | 1.35 (1.06–1.72) | 1.99 (1.52–2.61) | 2.10 (1.41–3.14) | < 0.001 | 1.59 (1.19–2.13) |  |
| Ever smoker |  |  |  |  |  |  | 0.080 |
| No (*n* = 367,550) | Reference | 1.58 (1.07–2.34) | 0.97 (0.54–1.75) | 2.53 (1.24–5.13) | 0.463 | 1.07 (0.56–2.06) |  |
| Yes (*n* = 264,119) | Reference | 1.36 (1.05–1.75) | 1.99 (1.49–2.66) | 2.15 (1.40–3.31) | < 0.001 | 1.68 (1.24–2.29) |  |

*CI,* confidence interval; *FBG,* fasting blood glucose; *HbA1c,* hemoglobin A1c.

Cox proportional hazard models were used with age as a timescale to estimate hazard ratios and 95% CIs. The multivariable model was adjusted for age (timescale); sex; center; screening year; smoking status; regular exercise; body mass index; education level; dyslipidemia medication use; history of hypertension, chronic obstructive pulmonary disease, and asthma; and family history of cancer.

^a^HbA1c < 5.7, 5.7–5.9, 6.0–6.4, and ≥6.5% correspond to < 39, 39–41, 42–46, and ≥48 mmol/mol, respectively.

^b^FBG < 90, 90–­99, 100­–125, and ≥126 mg/dL correspond to < 5.0, 5.0–5.5, 5.6–6.9, and ≥7.0 mmol/L, respectively.

**Supplementary Table S2** Hazard ratios (95% CIs)^a^ for lung cancer mortality by insulin resistance in clinically relevant subgroups

| **Subgroup** | **Insulin resistance** | | ***p* for interaction** |
| --- | --- | --- | --- |
|  | **HOMA-IR < 2.5** | **HOMA-IR ≥ 2.5** |  |
| **Age (years)** |  |  | 0.208 |
| < 50 (*n* = 549,133) | Reference | 1.76 (1.18–2.61) |  |
| ≥ 50 (*n* = 117,755) | Reference | 1.33 (1.07–1.65) |  |
| **Sex** |  |  | 0.720 |
| Female (*n* = 317,664) | Reference | 1.50 (1.00–2.25) |  |
| Male (*n* = 349,224) | Reference | 1.38 (1.11–1.72) |  |
| **Ever smoker** |  |  | 0.477 |
| No (*n* = 367,550) | Reference | 1.61 (1.11–2.35) |  |
| Yes (*n* = 264,119) | Reference | 1.38 (1.10–1.74) |  |

*CI,* confidence interval; *HOMA-IR,* homeostasis model assessment of insulin resistance.

^a^Cox proportional hazard models were used with age as a timescale to estimate hazard ratios and 95% CIs. The multivariable model was adjusted for age (timescale); sex; center; screening year; smoking status, regular exercise; body mass index; education level; dyslipidemia medication use; history of hypertension, chronic obstructive pulmonary disease, and asthma; and family history of cancer.

**Supplementary Table 3** Hazard ratios (95% CIs) for lung cancer mortality per glucose category in the overall population after excluding lung cancer mortality cases that occurred during the first 2–4 years of the follow-up period

|  | **Multivariable-adjusted HR^a^ (95% CI)** | | |
| --- | --- | --- | --- |
|  | **After excluding 54 cases occurring in the first 2 years of the follow-up period** | **After excluding 88 cases occurring in the first 3 years of the follow-up period** | **After excluding 137 cases occurring in the first 4 years of the follow-up period** |
| **HbA1c category^b^ (%)** |  |  |  |
| < 5.7 | 1.00 (reference) | 1.00 (reference) | 1.00 (reference) |
| 5.7–5.9 | 1.28 (1.02–1.60) | 1.25 (0.99–1.58) | 1.21 (0.95–1.54) |
| 6.0–6.4 | 1.63 (1.25–2.12) | 1.62 (1.24–2.13) | 1.51 (1.14–2.01) |
| ≥ 6.5 (screen-detected diabetes) | 2.19 (1.52–3.15) | 2.12 (1.45–3.10) | 1.93 (1.29–2.90) |
| P for trend | <0.001 | <0.001 | 0.001 |
| Previously diagnosed diabetes | 1.53 (1.16–2.03) | 1.61 (1.21–2.14) | 1.52 (1.13–2.05) |
| **FBG category^c^ (mg/dl)** |  |  |  |
| < 90 | 1.00 (reference) | 1.00 (reference) | 1.00 (reference) |
| 90–99 | 1.03 (0.81–1.32) | 1.04 (0.81–1.35) | 1.05 (0.80–1.37) |
| 100–125 | 1.31 (1.02–1.68) | 1.27 (0.98–1.65) | 1.32 (1.00–1.73) |
| ≥ 126 (screen-detected diabetes) | 1.80 (1.16–2.79) | 1.92 (1.23–2.98) | 1.77 (1.10–2.85) |
| P for trend | 0.002 | 0.002 | 0.005 |
| Previously diagnosed diabetes | 1.43 (1.04–1.97) | 1.51 (1.09–2.09) | 1.49 (1.06–2.10) |
| **Insulin resistance^d^** |  |  |  |
| HOMA-IR < 2.5 | 1.00 (reference) | 1.00 (reference) | 1.00 (reference) |
| HOMA-IR ≥ 2.5 | 1.47 (1.17–1.85) | 1.51 (1.20–1.91) | 1.52 (1.19–1.94) |

*CI,* confidence interval; *FBG,* fasting blood glucose; *HbA1c,* hemoglobin A1c; *HOMA-IR,* homeostasis model assessment of insulin resistance; *HR,* hazard ratio.

^a^Cox proportional hazard models were used with age as a timescale to estimate hazard ratios and 95 percent confidence intervals. The multivariable model was adjusted for age (timescale); sex; center; screening year; smoking status; regular exercise; body mass index; education level; dyslipidemia medication use; history of hypertension, chronic obstructive pulmonary disease, and asthma; and family history of cancer.

^b^HbA1c < 5.7, 5.7–5.9, 6.0–6.4, and ≥ 6.5% correspond to < 39, 39–41, 42–46, and ≥ 48 mmol/mol, respectively.

^c^FBG < 90, 90–­99, 100­–125, and ≥ 126 mg/dL correspond to < 5.0, 5.0–5.5, 5.6–6.9, and ≥ 7.0 mmol/L, respectively.

^d^Among subjects without previously diagnosed diabetes.

**Supplementary Table 4** Hazard ratios (95% CIs) for lung cancer mortality by glycemic status and duration of diabetes (*n* = 658,973)

|  | | **Person-years** | **Number of events** | **Mortality rate**  **(10^5^ PY)** | **Multivariable-adjusted HR^a^**  **(95% CI)** |
| --- | --- | --- | --- | --- | --- |
| **HbA1c category ^b^ (%)** | |  |  |  |  |
| < 5.7 | | 4,119,484 | 249 | 6.0 | 1.00 (reference) |
| 5.7–5.9 | | 1,027,740 | 148 | 14.4 | 1.37 (1.11–1.70) |
| 6.0–6.4 | | 303,919 | 92 | 30.3 | 1.69 (1.32–2.18) |
| ≥ 6.5 (screen-detected diabetes) | | 78,782 | 37 | 47.0 | 2.21 (1.55–3.15) |
| Previously diagnosed diabetes | |  |  |  |  |
| Duration of diabetes  (years) | < 5 | 43,638 | 12 | 27.5 | 1.66 (0.92–3.00) |
|  | 5-9.9 | 16,009 | 6 | 37.5 | 1.55 (0.68–3.52) |
|  | ≥ 10 | 13,805 | 5 | 36.2 | 0.98 (0.40–2.39) |
| **FBG category ^c^ (mg/dL)** | |  |  |  |  |
| < 90 | | 1,916,655 | 110 | 5.7 | 1.00 (reference) |
| 90–99 | | 2,428,299 | 190 | 7.8 | 1.05 (0.83–1.33) |
| 100–125 | | 1,112,086 | 200 | 18.0 | 1.38 (1.09–1.75) |
| ≥ 126 (screen-detected diabetes) | | 72,886 | 26 | 35.7 | 1.71 (1.11–2.64) |
| Previously diagnosed diabetes | |  |  |  |  |
| Duration of diabetes (years) | < 5 | 43,638 | 12 | 27.5 | 1.54 (0.84–2.83) |
|  | 5-9.9 | 16,009 | 6 | 37.5 | 1.41 (0.61–3.26) |
|  | ≥ 10 | 13,805 | 5 | 36.2 | 0.89 (0.36–2.20) |

*BMI,* body mass index; *CI,* confidence interval; *COPD,* chronic obstructive pulmonary disease; *FBG,* fasting blood glucose; *HbA1c,* hemoglobin A1c; *HR,* hazard ratio;

^a^Cox proportional hazard models were used with age as a timescale to estimate HRs and 95% CIs. The multivariable model was adjusted for age (timescale), sex, center, screening year, smoking status, regular exercise, BMI, education level, history of hypertension, dyslipidemia medication use, history of COPD, history of asthma, and family history of cancer.

^b^HbA1c < 5.7, 5.7–5.9, 6.0–6.4, and ≥ 6.5% corresponds to < 39, 39–41, 42–46, and ≥ 48 mmol/mol, respectively.

**Supplementary Table 5** Hazard ratios (95% CIs) for lung cancer mortality by glycemic status and age at diabetes diagnosis (*n* = 658,973)

|  | | **Person-years** | **Number of events** | **Mortality rate**  **(10^5^ PY)** | **Multivariable-adjusted HR^a^**  **(95% CI)** |
| --- | --- | --- | --- | --- | --- |
| **HbA1c category ^b^ (%)** | |  |  |  |  |
| < 5.7 | | 4,119,484 | 249 | 6.0 | 1.00 (reference) |
| 5.7–5.9 | | 1,027,740 | 148 | 14.4 | 1.37 (1.11–1.69) |
| 6.0–6.4 | | 303,919 | 92 | 30.3 | 1.69 (1.31–2.17) |
| ≥ 6.5 (screen-detected diabetes) | | 78,782 | 37 | 47.0 | 2.21 (1.55–3.15) |
| Previously diagnosed diabetes | |  |  |  |  |
| age at diabetes diagnosis  (years) | < 35 | 10,042 | 0 |  | - |
|  | 35-49.9 | 35,622 | 8 | 22.5 | 2.10 (1.03–4.28) |
|  | ≥ 50 | 27,788 | 15 | 54.0 | 1.25 (0.73–2.14) |
| **FBG category ^c^ (mg/dL)** | |  |  |  |  |
| < 90 | | 1,916,655 | 110 | 5.7 | 1.00 (reference) |
| 90–99 | | 2,428,299 | 190 | 7.8 | 1.05 (0.83–1.33) |
| 100–125 | | 1,112,086 | 200 | 18.0 | 1.38 (1.09–1.75) |
| ≥ 126 (screen-detected diabetes) | | 72,886 | 26 | 35.7 | 1.71 (1.11–2.64) |
| Previously diagnosed diabetes | |  |  |  |  |
| age at diabetes diagnosis (years) | < 35 | 10,042 | 0 |  | - |
|  | 35-49.9 | 35,622 | 8 | 22.5 | 2.00 (0.97–4.14) |
|  | ≥ 50 | 27,788 | 15 | 54.0 | 1.13 (0.65–1.97) |

*BMI,* body mass index; *CI,* confidence interval; *COPD,* chronic obstructive pulmonary disease; *FBG,* fasting blood glucose; *HbA1c,* hemoglobin A1c; *HR,* hazard ratio;

^a^Cox proportional hazard models were used with age as a timescale to estimate HRs and 95% CIs. The multivariable model was adjusted for age (timescale), sex, center, screening year, smoking status, regular exercise, BMI, education level, history of hypertension, dyslipidemia medication use, history of COPD, history of asthma, and family history of cancer.

^b^HbA1c < 5.7, 5.7–5.9, 6.0–6.4, and ≥ 6.5% corresponds to < 39, 39–41, 42–46, and ≥ 48 mmol/mol, respectively.

**Supplementary Table 6.** Hazard ratios (95% CIs) for lung cancer mortality by waist circumference (*n* = 562,111)

|  | **Person-years** | **Number of events** | **Mortality rate**  **(10^5^ PY)** | **Multivariable-adjusted HR^a^**  **(95% CI)** |
| --- | --- | --- | --- | --- |
| **Abdominal obesity**^b^ |  |  |  |  |
| No | 3,439,102 | 327 | 8.5 | 1.00 (reference) |
| Yes | 884,386 | 153 | 14.8 | 1.72 (1.34–2.22) |
| Per 1 cm increase in waist circumference |  |  |  | 1.05 (1.02–1.07) |

*BMI,* body mass index; *CI,* confidence interval; *COPD,* chronic obstructive pulmonary disease; *FBG,* fasting blood glucose; *HbA1c,* hemoglobin A1c; *HR,* hazard ratio;

^a^Cox proportional hazard models were used with age as a timescale to estimate HRs and 95% CIs. The multivariable model was adjusted for age (timescale), sex, center, screening year, smoking status, regular exercise, BMI, education level, history of hypertension, dyslipidemia medication use, history of COPD, history of asthma, and family history of cancer.

^b^For men, abdominal obesity by waist circumference ≥ 90 cm; for women, abdominal obesity by waist circumference ≥ 85 cm.
